# Applying Multi-modal and Correlation Analysis on Environmental Parameters and Effect on Cardiopulmonary Endurance of Gender in Elderly People

**Published:** 2018-04

**Authors:** Sungju LEE, Mehdi REZAEI, Taikyeong JEONG

**Affiliations:** 1. Dept. of Computer Convergence Software, Korea University, Seoul, Republic of Korea; 2. College of Business, CHA University, Gyeonggi-do, Republic of Korea

**Keywords:** Multi-modal analysis, Correlation analysis, Cardiopulmonary endurance, Air pollution

## Abstract

**Background:**

The aim of this study was to investigate the correlation and interaction between the air pollution’s components with cardiopulmonary endurance of elderly people in eight regions by using a multi-modal and correlation analysis.

**Methods:**

The data of air pollution was collected in eight selected regions in 2013 to 2015. At the same time, total number of 880 male and female, older than 65 year-olds, were investigated based on the cardiopulmonary endurance measurement in the same regions. The correlation, interaction and multiple linear regressions was tested between the air pollution components in each region and cardiopulmonary endurance of elderly people, also between the air pollution components in each region and gender, respectively. In this case, the regression analysis for both hypotheses was conducted.

**Results:**

There was a correlation between the level of air pollution and cardiopulmonary endurance, especially for the carbon monoxide which has a strong effect, it was followed by the effect of sulfur dioxide and fine dust, meanwhile nitrogen dioxide seems not to be effective for this measurement test. Furthermore, it was highly unlikely that gender was a significant factor for the correlation between air pollution and cardiopulmonary endurance.

**Conclusion:**

The importance and correlation between air pollution and cardiopulmonary capacity is a critical determinant for the public health of a society, while at the same time having a serious impact on certain age groups. Provided that the factor of gender is highly unlikely to modify this impact, it is necessary to study the potential of other factors.

## Introduction

The cardiopulmonary endurance of an individual is determined by many factors, including both lifestyle choices, such as exercise and obesity (1, 2), as well as environmental conditions, like air pollution. For patients with heart failure, exposure to air pollution was shown to be a determinant of mitigating the health benefits of exercise (3, 4) In fact, the rate of pollution will negatively affect the exercise capacity of this group of patients (5).

Ample research claimed on the association between the air pollution and cardiovascular diseases (6–11). The association between the ambient particulate matter and the increased risk of cardiovascular outcomes was consistently shown by previous studies (12). In particular, the amount of fine dust in the atmosphere is an important issue with respect to problems of human and the associated health management (13, 14). Meanwhile it is not still uncertainty as to whether the physical exercise in an environment with substantial air pollution will be harmful to the health or not (15), it is clear that there is statistically and practically a significant correlation between the air pollution and the health of human (6–11).

The harmful impact of environmental data on the health has been shown to be affected by several factors, such as age (15, 16) or activity by people (17, 18). Provided that the demographic trend of many countries, including South Korea (19), is toward an older population, and also the atmospheric information of the recent years presents higher level of fine dust and CO_2_ in this country (20), environmental factors potentially could be considered as harmful for the health of elderly people in South Korea.

Accordingly, this study aimed to investigate the correlations and interactions between the air pollution and cardiopulmonary endurance of elderly people in South Korea. Employing the multi correlation analysis, we aimed to explore those factors that potentially could modify this correlation.

## Methods

### Study area

This study was conducted in eight regions located in South Korea. In order to determine the atmospheric data of each area, the following components were collected, during 2013–2015, Fine dust (mg/m^3^), nitrogen dioxide (ppm), carbon monoxide (ppm), and sulfur dioxide (ppm). The areas with the highest levels of air pollution were Gyeonggi and Jeonbuk, while Gwangju and Chungnam showed relatively low levels of air pollution.

### Sample study

The sample group of this study was the elderly people (aged 65 and above) living in the mentioned regions of South Korea. In 2015, we measured the cardiopulmonary endurance of elderly people living in each of the eight aforementioned regions, by recording the total distance moving by an individual after six-minutes of walking. The six-minute walk test is a standard measure of cardiopulmonary endurance that provides an assessment of the physical strength of the elderly (21). A total of 880 data points were collected across all regions, Seoul collected 180 male and female elderly data, and 100 male and female elderly data collected elsewhere.

Informed consent was taken from the participants and the study was approved by the Ethics Committee of the university.

### Statistical analysis

It was hypothesized that there is a strong interaction between genders on the result of cardiopulmonary endurance exercise test and regions, and also the gender with air pollution. Responding these hypotheses, we analyzed the correlations and interactions of atmospheric pollution and the cardiopulmonary endurance by statistical multi-model relations.

The interaction was tested based on the explanatory variables and response variables. In order to this, our experimental setup was to 1-test the subject effects of each independent variable which are the components of air pollution on the CE as dependent variable 2-introducing gender to the model and testing their effect on CE with eight different analysis (4 for male and 4 for female), 3-testing the interaction of air pollution and gender on the CE.

The analysis was designed and conducted based on two main models, including: i) the correlation between air pollution in each regain and cardiopulmonary endurance measure. ii) The interaction between air pollution in each regain and the cardiopulmonary endurance based on gender. All analysis was conducted by a statistical software tool, SPSS ver. 22 (Chicago, IL, USA).

## Results

Based on the first model (the correlation between air pollution in each regain and cardiopulmonary endurance measure) we found significant correlation between air pollution and CE, also the results of the analysis showed that carbon monoxide has the greatest effect on the cardiopulmonary endurance of the elderly, followed by the presence of fine dust and sulfur dioxide (Table 1). The correlation analysis showed that the correlation coefficient of fine dust, nitrogen dioxide, carbon monoxide, and sulfur dioxide with the cardiopulmonary endurance of the elderly was 0.68 (i.e., male 0.62, female 0.73), 0.15 (*i.e.*, male 0.15, female 0.14), 0.89 (*i.e.*, male 0.91, female 0.85), and 0.61 (*i.e.*, male 0.67, female 0.54), respectively.

**Table 1:** Statistical Analysis of the measured data CE test, Air pollution with region

***Classification***	***Air conditions with regions in 2013–2015***				***Cardiopulmonary endurance measurement age 65 to 97 years***			

**Regions**	**Fine dust (mg/m^3^)**	**Nitrogen dioxide (ppm)**	**Carbon monoxide (ppm)**	**Sulfur dioxide (ppm)**	**Male (0.1m/6min)**	**Female (0.1m/6min)**		**Total (0.1m/6min)**
Seoul	45.33	0.03	0.50	0.01	537.83 ± 232.60	538.61 ± 221.63		538.22 ± 283.28
Incheon	49.00	0.03	0.60	0.01	357.28 ± 240.71	383.00 ± 266.75		370.14 ± 284.15
Gwangju	41.67	0.02	0.50	0.00	523.64 ± 219.52	510.86 ± 242.21		517.25 ± 250.20
Gyeonggi	54.00	0.03	0.60	0.01	420.59 ± 227.60	393.47 ± 225.11		407.03 ± 231.09
Chungnam	42.00	0.02	0.50	0.00	511.24 ± 257.62	521.97 ± 243.33		516.60 ± 256.11
Jeonbuk	51.00	0.02	0.50	0.00	471.07 ± 225.67	440.03 ± 243.67		455.55 ± 285.89
Gyeongbuk	49.67	0.02	0.60	0.01	405.56 ± 223.34	427.04 ± 215.83		416.30 ± 223.10
Gyeongnam	47.33	0.02	0.50	0.00	553.35 ± 202.51	559.96 ± 228.57		556.66 ± 207.51
	**Correlation Analysis**				**Regression Analysis**			
Male	0.62	0.15	0.91	0.67	R^2^	0.8635	*P*-value	0.0093
Female	0.73	0.14	0.85	0.54	R^2^	0.7740	*P*-value	0.0006
Total	0.68	0.15	0.89	0.61	R^2^	0.8113	*P*-value	0.0004

For the correlation analysis and determination of effect size, the air pollution data was combined by averaging the measured data for each factor. There was a strong, positive correlation between the amount of carbon monoxide in the air and the cardiopulmonary endurance of the elderly. A positive correlation was also found between the amount of fine dust and sulfur dioxide present in the atmosphere and the cardiopulmonary endurance of the elderly. Relative to the group, nitrogen dioxide showed a weak, positive correlation. Additionally, it was confirmed that males with outdoor lifestyles correlated more strongly than females.

Finally, a multiple regression analysis was used to examine the correlation between the four air pollution factors and the cardiopulmonary endurance of the elderly. As a result of the analysis, we confirmed that the presence of air pollution has a very strong, positive effect on the cardiopulmonary endurance of the elderly, as indicated by a *P*-value <0.05 using a coefficient of determination (*i.e.*, R^2^) of 0.81 (*i.e.*, male 0.86, female 0.77).

**Fig. 1:**
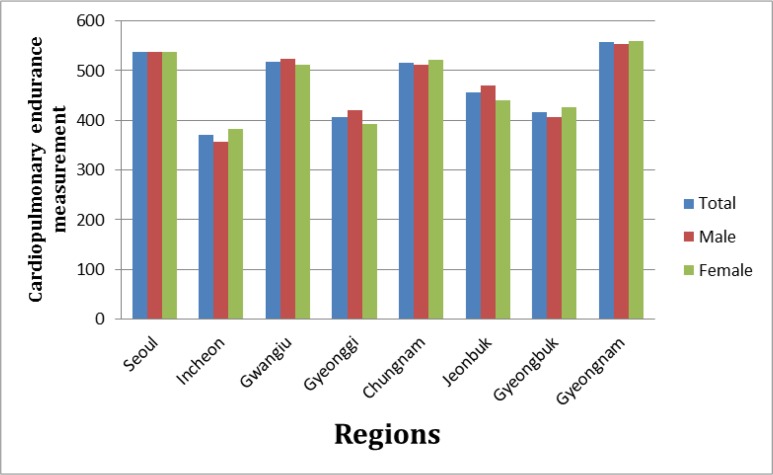
Result of Cardiopulmonary endurance (CE) exercise test based on gender in case study area

As it is presented in Fig. 1, the distribution of CE result test is shown based on the genders in each region. It is very clear that the level of CE result test in Seoul and Gyeongnam are the highest rates comparing with the other areas, it is followed by Chungnam and Gwangju. However, Incheon is a city with the lowest rate of CE.

Accordingly to the second model (The interaction between air pollution in each regain and the cardiopulmonary endurance based on gender), it was hypothesized that the level of air pollution in each region has a different proportion for male and female.

In order to address this question by quantitative analysis, we have tested the interactions between the gender and each region, also between gender and the air pollution in each region. An independent sample, T test, was employed in order to compare the average of cardiopulmonary endurance measure for male and female in each region. Based on the results presented in Table 2, it seems that there is not a significant differences based on the gender. For instance, the *P*-value of Seoul is not significant at the rate of 0.9817 (>0.05). Accordingly, we report that the performance of men and female body after the six min. exercise test was equivalent in all areas.

**Table 2:** T test results for gender with CE test based on regions

***Regions***	**P-*value***
Seoul	0.9817
Incheon	0.6134
Gwangju	0.7827
Gyeonggi	0.5505
Chungnam	0.8307
Jeonbuk	0.5102
Gyeongbuk	0.8785

On the other hand, we also considered the interaction of CE proportion for both genders and air pollution factors. In order to address these test, we used Multiple Linear Regressions analyses which means fitting a linear equation to the observed data and making a model based on the relationship among them. The interaction is being tested based on the explanatory variables and response variables. The conducted tests are shown in Table 3. The factor of gender, failed to present a significant impact on CE, when the effect of air pollutions was adjusting, (*P*. value >0.05) for Fine dust 0.842, Nitrogen dioxide 0.982, Carbon monoxide 0.753 and Sulfur dioxide 0.751. Testing the integration of each air pollution representative and gender, showed that by increasing the level of air pollution both genders will be affected similarly; based on the *P*. values of 0.840, 0.985, 0.755 and 0.750 for the Fine dust, Nitrogen dioxide, Carbon monoxide and Sulfur dioxide, respectively. It is clear that none of these tests is significant which implies on this fact that although the air pollution is significant for CE level, there is not a significant difference based on the gender.

**Table 3:** Effect and Interaction of air pollution and gender based on the Multiple Linear Regressions

***Factors***	***Effect of Sex (Gender)***		***Interaction by Sex (Gender)***	
	**F statistic**	**P-Value**	**F statistic**	**P-Value**
Fine dust (10mgm^3^)	0.042	0.842	0.043	0.840
Nitrogen dioxide (ppm)	0.001	0.982	<.001	0.985
Carbon monoxide (ppm)	0.104	0.753	0.102	0.755
Sulfur dioxide (ppm)	0.105	0.751	0.106	0.750

Degree of freedom of all F tests were F (1,12)

## Discussion

In this study, data on the atmospheric environment and cardiopulmonary endurance of the elderly people living in eight region of South Korea, was collected and analyzed based on the objective that was finding their potential correlations. The results of the analysis showed that carbon monoxide has the greatest effect on the cardiopulmonary endurance of the elderly, followed by the presence of fine dust and sulfur dioxide. Considering elderly people, their cardiopulmonary endurance measurement is correlated with air pollution, meanwhile this correlation is unlikely to differ based on their gender.

While a study reported that there was not a significant relationship between particulate matters and elderly people elderly (22), another study conducted in United States reported a positive association among sensitive subgroups (16). Similarly we found that elderly people are affected by air pollution. Furthermore, it was asserted that it might be due to some determinant, for instance, the higher level of air pollution and also the time being exposure to that (16). It is likely that the gender and the components of air pollution have an equal interaction for male and female. In other word, it implies on this fact that when there is an increase in the proportion of air pollution, the result of CE test of elderly people will differ; meanwhile there will be almost a similar effect on both genders.

Air pollution is not only a contributor for respiratory and cardiovascular diseases which will lead to mortalities (6–11), it also plays a significant role for people with certain age group, for instance elderly people. Therefore, the importance of correlation and cardiopulmonary capacity is a critical concern of public health in a society.

Given that protecting the general health of people is the main concern of all humane society (23), the implication of this study would be helpful for the associated authorities. A suggested remedy for people with heart failure who are exposure to diesel engine exhaust was reported to be simple respiratory filters which can adverse effects of pollution (5). Elderly people will face a better health regarding CE, in the case that the government and the associated organizations carefully monitor the amount of carbon monoxide available in the air of South Korea.

## Conclusion

Controlling the amount of air pollution, particularly Carbon monoxide, will affect the CE level among elderly people living in South Korea, meanwhile the factor of gender is unlikely to play a role in modifying this impact. Therefore, our results are indicated that a multi-modal and correlation analysis is highly related to environmental parameters such as cardiopulmonary endurance of gender in elderly people. In order to complete the multiple statistical analyses, several environmental parameters are used and investigated based on CE, gender, ages, and regions, etc.

## Ethical considerations

Ethical issues (Including plagiarism, informed consent, misconduct, data fabrication and/or falsification, double publication and/or submission, redundancy, etc.) have been completely observed by the authors.
